# Supplementary material for: Can digital financial inclusion converge the regional agricultural carbon emissions intensity gap?
Source: PLoS One. 2024 Jul 16;19(7):e0307328. doi: 10.1371/journal.pone.0307328 (PMC11251634; doi:10.1371/journal.pone.0307328)
Supplement: Attachment — Submitted filename: Response to Reviewers.docx [file pone.0307328.s001.docx]

Dear Editors and Reviewers:

Thank you for your letter and for the reviewers’ comments concerning our manuscript entitled “Can digital financial inclusion converge the regional agricultural carbon emissions gap?” Those comments are all valuable for revising and improving our paper, as well as in favor of guiding our research.

We have accepted the reviewers’ important recommendations and strive for the best efforts to improve the manuscript. Specifically, we have made some revisions to the manuscript, but these changes will not affect the main content and framework of this paper. Revised portion are marked with red fonts in the paper. The main corrections in the paper and the responds to the reviewers’ comments are as flowing.

We appreciate for Editors/Reviewers’ warm work earnestly, and hope that the correction will meet with approval.

Once again, thank you very much for your comments and suggestions.

Sincerely yours,

Lingzhi Tan

**Responds to the Editor's comments:**

**1.	Please ensure that your manuscript meets PLOS ONE's style requirements, including those for file naming. The PLOS ONE style templates can be found at**

**https://journals.plos.org/plosone/s/file?id=wjVg/PLOSOne_formatting_sample_main_body.pdf and**

**https://journals.plos.org/plosone/s/file?id=ba62/PLOSOne_formatting_sample_title_authors_affiliations.pdf:** Thanks for the editor's comments. We apologize for the formatting errors in the initial draft. At the time of submission, we only had access to one file outlining the formatting requirements, which led to the errors. We have since made revisions to the draft in strict accordance with the journal's formatting standards.

**2.	Please state what role the funders took in the study. Please include this amended Role of Funder statement in your cover letter; we will change the online submission form on your behalf.**

**Response:** Thanks for the editor's comments. We have modified and added this section to the cover letter, as shown in the attached document.

**3.	Please remove any funding-related text from the manuscript and let us know how you would like to update your Funding Statement.** **Please include your amended statements within your cover letter.**

**Response:** Thanks for the editor's comments. We have removed any funding-related text from the manuscript. And we have updated our funding statement in the cover letter, as shown in the attached document.

**4.	Please ensure that you have an ORCID iD and that it is validated in Editorial Manager.**

**Response:** Thanks for the editor's comments. Apologies for our oversight, the ORCID ID has now been updated.

**5.	Please review your reference list to ensure that it is complete and correct.**

Thanks for the editor's comments. After examining the literature, we found that none of the references in the manuscript had been retracted. However, we suspect that the issue raised by the editor may be due to the fact that several of the cited documents are in Chinese. Therefore, we have updated some of the references in the paper to incorporate their content.

**The corresponding contents of the revised manuscript are as follows:**

Pang J, Li H, Lu C, Lu C, Chen X. Regional Differences and Dynamic Evolution of Carbon Emission Intensity of Agriculture Production in China. International Journal of Environmental Research and Public Health. 2020; 17(20):7541. [doi:10.3390/ijerph17207541](https://doi.org/10.3390/ijerph17207541)

Wang L, Tang J, Tang M, Su M, Guo L. Scale of Operation, Financial Support, and Agricultural Green Total Factor Productivity: Evidence from China. Int J Environ Res Public Health. 2022;19(15):9043. Published 2022 Jul 25. doi:10.3390/ijerph19159043

Zhang F, Deng X, Phillips F, Fang CL, Wang C. Impacts of industrial structure and technical progress on carbon emission intensity: Evidence from 281 cities in China. Technological Forecasting & Social Change. 2020; 154:435-447.[doi: 10.1016/j.techfore.2020.119949](https://doi.org/10.1016/j.techfore.2020.119949)

Wei P, Xiong LY. Managing financing costs and fostering green transition: The role of green financial policy in China. Economic Analysis and Policy.2022;76:820-836. doi: 10.1016/J.EAP.2022.09.014

Lyu XY. The Impact of the Digital Economy on Carbon Emissions: Evidence from China. Journal of Empirical Studies. 2022; 9:15-23. doi:10.18488/66. v9i1.3066.

Hong M, Tian M, Wang J. Digital financial inclusion, Agricultural Industrial Structure Optimization and Agricultural Green Total Factor Productivity. Sustainability. 2022; 14(18):11450. doi: 10.3390/su141811450

Sun YN, You XT. Do digital financial inclusion, innovation, and entrepreneurship activities stimulate vitality of the urban economy? Empirical evidence from the Yangtze River Delta, China. Technology in Society. 2023; 72. doi: 10.1016/j.techsoc.2023.102200

Liu Y, Luan L, Wu WL, Zhang ZQ, Hsu Y. Can digital financial inclusion promote China's economic growth? International Review of Financial Analysis.2021;78:101889. doi: 10.1016/J.IRFA.2021.101889

Wang WJ, He TY, Li ZH. Digital financial inclusion, economic growth and innovative development. Kybernetes. 2022. doi:10.1108/K-09-2021-0866.

**6.	Please ensure that you refer to Figure 1 in your text as, if accepted, production will need this reference to link the reader to the figure.**

**Response:** Thanks for the editor's comments. We have re-referenced Figure 1 in the text.

**Responds to the reviewer’s comments:**

**Reviewers’ comments:**

**Reviewer #1: Agricultural carbon intensity would be analyses with the quantile method in this article. I would suggest publishing the article as it is because it is provided sufficient evidence to investigate the occasion.**

**Response:** Thanks for the reviewer’s comments. It is our honor to receive your approval.

**Reviewer #2:**

**1. Introduction: please start your elaboration from a much more macro perspective or background; for instance, you can illustrate the importance in a global background rather than only China.**

**Response:** Thanks for the reviewer’s comments. Your feedback was very helpful, as it specifically highlighted issues with the introduction section of our paper. As a result, we have made revisions to the introduction in order to emphasize the global significance of the topic, rather than solely focusing on China.

**The corresponding contents of the revised manuscript are as follows:**

The former establishment of a green, low-carbon, and sustainable global governance system has become a common development goal for all countries. Agricultural production is strongly affected by and a major contributor to climate change. Agriculture and land-use change account for a quarter of total global emissions of greenhouse gases (GHG) ^[1]^. In the 20-year period, China was responsible for the most emissions from agricultural production ^[2]^. Consequently, developing LCA with "high efficiency, low energy consumption, low emissions, and high carbon sinks" is crucial.

**2. You should update your references.**

**Response:** Thanks for the reviewer’s comments. We have read the references provided by you and found them to be very useful in enhancing our paper. Therefore, we have cited all of them in the manuscript. For details, please refer to the revised manuscript.

**3. Redraw figure 1: some lines seem wrong or improper.**

**Response:** Thanks for the reviewer’s comments. We identified some issues with Figure 1 and have reworked it accordingly.

**The corresponding contents of the revised manuscript are as follows:**

**Fig 1. Schematic diagram of the research hypothesis**

**4. Should model setting be placed before the descriptive statistical analysis? Please check whether current order is proper.**

**Response:** Thanks for the reviewer’s comments. We have placed the model setting before the descriptive statistics analysis. For details, please refer to the revised manuscript.

**5. Refine titles. Some titles are too long; some are not proper; for instance, in 5.4, “etc.” is not commonly used in titles.**

**Response:** Thanks for the reviewer’s comments. We reread the article and made simplifying changes to the headline.

**The corresponding contents of the revised manuscript are as follows:**

2.3 Mechanism of action

2.4 Moderating effect

4.3 Mechanism analysis

4.4 Moderating effect

5.4 Decomposition of interregional disparities

6. Conclusions and countermeasures

6.1 Conclusions

6.2 Countermeasures

**6. Section 6: put implications first, and then put conclusions. Also demonstrate your novelties and limitations.**

**Response:** Thanks for the reviewer's comments. We have revised the Section 6 by first presenting the implications, followed by the conclusions and countermeasures. Additionally, we have included a discussion of the novelties and limitations of our paper at the end.

**The corresponding contents of the revised manuscript are as follows:**

This article analyzes the impact and mechanism of inclusive finance on the agricultural carbon emission intensity value and regional carbon emission intensity D-Gini coefficient of 30 sample provinces from 2010 to 2020. The study can provide relevant policy support for the current stage of digital inclusion financial to reduce agricultural carbon emissions and improve digitalinclusive finance support for the transformation and upgrading of the regional agricultural industry, as well as provide feasible experiences for other countries. We get the following key conclusions in this study:

6.3 Innovations and limitations

The innovations in this study are as follows.

1. It analyzes the impact of digital financial inclusion on agricultural carbon emission reduction intensity to determine whether it can help reduce agricultural carbon emissions and narrow the gap in agricultural carbon emission intensity between regions.

2. It explains the impact mechanism of digital financial inclusion from the perspectives of financial resource allocation capacity and technological progress, providing a new perspective for analyzing the path of convergence of the regional carbon emission intensity gap through digital financial inclusion.

3. It introduces the urban-rural income gap and provides an in-depth analysis of how digital financial inclusion affects the gap in agricultural carbon emission reduction intensity from the perspective of changes in the urban-rural income gap.

However, we also admit some limitations.

1. Limited by the research data, this paper only analyses the value of agricultural carbon emission intensity and regional carbon emission intensity D-Gini coefficient from 2010 to 2020, the results of digital financial inclusion on the two and the mechanism of action. Therefore, the timeliness of the data may be insufficient, and it is hoped that further research will be conducted in the future using updated data sources.

2. We cited more Chinese literature, mainly considering that this academic language has extensively studied issues related to this article, but documents in other languages may also be beneficial.

**7.** **Language must be polished. Current demonstration is Chinglish.**

**Response:** Thanks for the reviewer's comments. We have polished the language of the article and hope that the issues you raised will be improved. For details, please refer to the revised manuscript.
