# Supplementary material for: Can digital financial inclusion converge the regional agricultural carbon emissions intensity gap?
Source: PLoS One. 2024 Jul 16;19(7):e0307328. doi: 10.1371/journal.pone.0307328 (PMC11251634; doi:10.1371/journal.pone.0307328)
Supplement: Attachment — Submitted filename: Response to Reviewers.docx [file pone.0307328.s002.docx]

Dear Editors and Reviewers:

Thank you for your letter and the reviewers' comments concerning our manuscript entitled "Can digital financial inclusion converge the regional agricultural carbon emissions intensity gap?" Those comments are all valuable for revising and improving our paper and help guide our research.

We have accepted the reviewers' important recommendations and strive to make the best effort to improve the manuscript. Specifically, we have made some revisions to the manuscript, but these changes will not affect this paper's leading content and framework. Revised portions are marked with red fonts in the paper. The main corrections in the paper and the responds to the reviewers' comments are as flowing.

We appreciate for Editors/Reviewers' warm work earnestly, and hope that the correction will meet with approval.

Once again, thank you very much for your comments and suggestions.

Sincerely yours,

Lingzhi Tan

**Responds to the reviewer's comments:**

**Reviewers' comments:**

**Reviewer #3:**

**1. The reference style needs to be corrected to focus only on the family name. Additionally, all citations in the text should be updated in the reference.**

**Response:** Thanks for the reviewer's comments. We have also noticed this problem. Therefore, we have updated the references in a timely manner according to the journal formatting requirements. At the same time, all citations in the text were updated in the references accordingly.

**2. There are many typos throughout the paper, and the English needs to be polished. The authors sometimes use past tense and sometimes present tense, so this needs to be revised.**

**Response:** Thank you for your suggestions. They help us a lot to improve the quality of our articles. We have re-polished the language of the article.

**3. In the abstract, "D-GINI" should be written as the full word at the beginning where it first appears.**

**Response:** Thanks for the reviewer's comments. This was indeed an oversight, and we have now changed the first occurrence of the word "D-GINI" to the whole word.

**3. The contribution and research gap in this study needs to be mentioned in the last paragraph to highlight the novelty of the paper.**

**Response:** Thanks for the reviewer's comments. Section 6.3 of the article presents the contribution and research gap in this study as follows:

The innovations in this study are as follows.

1. It examines the impact of digital financial inclusion on the intensity of agricultural carbon emission reduction. It investigates whether it can facilitate the reduction of agricultural carbon emissions and the convergence of the regional gap in agricultural carbon emission intensity.

2. It elucidates the impact mechanism of digital financial inclusion from financial resource allocation efficiency and technological innovation, offering a novel perspective for analyzing the path of convergence of the regional gap in carbon emission intensity through digital financial inclusion.

3. It incorporates the urban-rural income disparity. It comprehensively analyzes how digital financial inclusion influences the gap in agricultural carbon emission reduction intensity from the perspective of changes in the urban-rural income disparity. However, we also acknowledge some limitations.

However, we also admit limitation: Due to the data availability, this paper only analyzes the values of agricultural and regional carbon emission intensity Dagum Gini coefficient from 2010 to 2020, the effects of digital financial inclusion on them, and the underlying mechanisms. Therefore, the timeliness of the data may be inadequate, and we hope that future research will be carried out with more updated data sources. So, the time series of data can be added in future research to enhance the effectiveness of the results.

**5. The term "digital inclusive finance" is mentioned in the abstract but only once in the literature review. It is unclear if "digital financial inclusion" and "digital inclusive finance" are the same thing or not. The authors should clarify this and also consider focusing on digital inclusive finance and CO2 emissions.**

**Response:** Thanks for the reviewer's comments. The issue you mentioned was an oversight in our writing and has now been changed to "digital financial inclusion."

**6. There are errors in lines 324, 496, and 528.**

**Response:** Thanks for the reviewer's comments. Many thanks to the reviewers for their meticulous review, which will immensely help our article. The errors have now been corrected and can be viewed in the main text.

**7. The Degum Gini coefficient is not explained in section 3.1 and needs to be defined.**

**Response:** Thanks for the reviewer's comments. The Degum Gini coefficient has been explained in Section 3.1 by defining it as follows:

In addition, the Dagum method was used to measure the differences in agricultural carbon emission intensity between regions in China. At this stage, the indicators for studying provincial differences mainly include the coefficient of variation, Mean Log Deviation Index (MLD), Tyrell's index, and Gini coefficient. However, when analyzing the above methods, most of them cannot decompose the provincial differences or do not consider the distribution of sample subgroups when deteriorating, so they all have certain defects in the application. To overcome the above difficulties, some researchers have used Dagum Gini coefficient to analyze provincial dynamic differences and evolutionary trends. In this paper, the Dagum Gini coefficient is utilized to measure the inter-provincial differences in the intensity of agricultural carbon emissions in China and decompose them to obtain the composition of the relative differences between inter-provincial regions.

$G=\sum_{j=1}^{k} \sum_{h=1}^{k} \sum_{i=1}^{C_{j}} \sum_{r=1}^{C_{h}} |y_{ji}-y_{hr}|/2C^{2}\bar{y}$（6）

In the equation, y_ji_ (y_hr_) denotes the agricultural carbon intensity of any provincial unit within the j(h) region, C is the number of sample provinces, is the national average of agricultural carbon intensity, k is the number of regional divisions, and C_j_(C_h_) is the number of provincial units within the j(h) region.

$G_{jh}=\sum_{i=1}^{G_{j}} \sum_{r=1}^{G_{h}} |y_{ji}-y_{hr}|/C_{j}C_{h}(\bar{Y}_{j}+\bar{Y}_{h})$（7）

Equation (7) is the Dagum Gini coefficient of agricultural carbon intensity between provinces j and h. $\bar{Y}_{j}$ and $\bar{Y}_{h}$ are the average agricultural carbon emission values of provinces j and h.

**7. The methodology section is problematic as it mixes up everything and is not explained clearly.**

**Response:** Thanks for the reviewer's comments. The method formulas utilized in the text are shown in the hope of answering your questions. The method in the text is specifically:

1.Baseline model

According to the research objectives and previous literature, we constructed the following RIF regression model:

$RIF\left\{ {lny}_{it},v^{D-G}(F_{Y}) \right\}=\beta_{0}+\beta_{1}X_{it-1}+\beta_{2}Z_{it-1}+\emptyset+\eta+\varepsilon_{it}$（1）

$RIF\left( lny_{it};q_{r},F \right)=q_{r}+\frac{\tau-I(lny_{it-1}\leq q_{r})}{f_{lny}(q_{r})}$（2）

$RIF\left( lny_{it} | \emptyset;\gamma;q_{r} \right)=B\tilde{X}\tilde{Z|}\emptyset+\varepsilon_{it}$（3）

To verify the establishment of H1, the base regression model adopts the regression method of regrouping influence function (RIF), as shown in Eq. (1); to consider the changes in the intensity of interprovincial agricultural carbon emission reductions at different quartiles, an unconditional quantile regression model is constructed based on the decentralized mapping function shown in Eq. (2), as shown in Eq. (3). In Eqs. (1)-(3), ${lny}_{it}$ denotes the value of agricultural carbon intensity in region i in year t, and $lny_{it-1}$ is the value of lagged one-period carbon intensity. The term $v^{D-G}(F_{Y})$ indicates the Dagum Gini coefficient of $lny$, which reflects the degree of imbalance in carbon intensity in province i in year t. X represents the digital financial inclusion index, further divided into the breadth of coverage, depth of use, and degree of digital services. $X_{it-1}$ is the lagged one-period index value for the sample provinces. $\beta_{0}$ is the intercept term, $\beta_{1}$and $\beta_{2}$ are the regression coefficient and $Z_{it-1}$ is a set of macro control variables lagged by one period i denotes the sample province, and t represents the year. $\emptyset$ signifies province and industry fixed effects, η refers to time-fixed effects, and $\varepsilon_{it}$ is a random disturbance term. $\tau$ represents quantile points, $q_{r}$ is the unconditional quantile function of $lny$, $f_{lny}(q_{r})$ is the probability density function, and B is the vector matrix of regression coefficients. $\tilde{X}$and $\tilde{Z}$ are the vector matrices of the core explanatory and control variables.

2. Calculation of carbon intensity in agriculture

Referring to the 2006 National Greenhouse Gas Inventory Guidelines Carbon Emission Factor Methodology recommended by the IPCC:

$TC=\sum C_{i}=\sum S_{i}\times\rho_{i}$（4）

Where TC is the total agricultural carbon emissions, $C_{i}$ denotes the total agricultural carbon emissions of the ith carbon emission source, $S_{i}$ refers to the total carbon emission input of the ith carbon emission source, and $\rho_{i}$ represents the emission factor of each carbon emission source. The reference coefficients and literature sources used in the equation are presented in Table 2.

**Table 2 Carbon emission factors for primary carbon sources**

| Carbon source factor | Carbon emission factor | Literature sources |
| --- | --- | --- |
| Fertilizer | 0.896 kgC .kg^-1^ | Oak Ridge National Laboratory |
| Pesticides | 4.934 kgC .kg^-1^ | Oak Ridge National Laboratory |
| Agricultural film | 5.180 kgC .kg^-1^ | The Institute of Resource, Ecosystem, and Environment of Agriculture (IREEA) of Nanjing Agricultural University |
| Diesel | 0.593 kgC.kg^-1^ | Intergovernmental Panel on Climate Change |
| Ploughing | 312.603 kgC.kg^-1^ | Wu FL et al.（2008）[50] |
| Irrigation | 20.476 kgC.kg^-1^ | Duan HP et al.（2012）[51] |

Calculate the value of agricultural carbon intensity according to equation (4):

$E_{agri}={TC}_{it}|{GDP}_{it}$（5）

In the equation, $E_{agri}$ represents the value of agricultural carbon emission intensity (KG/10000). GDP refers to the total agricultural output value of the sample provinces, and both variables are included in the equation using logarithmic values.

3. Calculation of inter-provincial differences in agricultural carbon intensity

In addition, the Dagum method was used to measure the differences in agricultural carbon emission intensity between regions in China. At this stage, the indicators for studying provincial differences mainly include the coefficient of variation, Mean Log Deviation Index (MLD), Tyrell's index Gini coefficient, etc. However, when analyzing the above methods, most of them cannot decompose the provincial differences or do not consider the distribution of sample subgroups when deteriorating, so they all have certain defects in the application. To overcome the above difficulties, some researchers have used Dagum Gini coefficient to analyze provincial dynamic differences and evolutionary trends. In this paper, the Dagum Gini coefficient is utilized to measure the inter-provincial differences in the intensity of agricultural carbon emissions in China and decompose them to obtain the composition of the relative differences between inter-provincial regions.

$G=\sum_{j=1}^{k} \sum_{h=1}^{k} \sum_{i=1}^{C_{j}} \sum_{r=1}^{C_{h}} |y_{ji}-y_{hr}|/2C^{2}\bar{y}$（6）

In the equation, y_ji_ (y_hr_) denotes the agricultural carbon intensity of any provincial unit within the j(h) region, C is the number of sample provinces, is the national average of agricultural carbon intensity, k is the number of regional divisions, and C_j_(C_h_) is the number of provincial units within the j(h) region.

$G_{jh}=\sum_{i=1}^{G_{j}} \sum_{r=1}^{G_{h}} |y_{ji}-y_{hr}|/C_{j}C_{h}(\bar{Y}_{j}+\bar{Y}_{h})$（7）

Equation (7) is the Dagum Gini coefficient of agricultural carbon intensity between provinces j and h. $\bar{Y}_{j}$ and $\bar{Y}_{h}$ are the average agricultural carbon emission values of provinces j and h.

**9.** **Eqs (1-3) in the methodology section are not clear, and the authors should explain whether they represent** **the same model and why they have different structures.**

**Response:** Thanks for the reviewer's comments. To verify the establishment of H1, the base regression model adopts the regression method of regrouping influence function (RIF), as shown in Eq. (1); to consider the changes in the intensity of interprovincial agricultural carbon emission reductions at different quartiles, an unconditional quantile regression model is constructed based on the decentralized mapping function shown in Eq. (2), as shown in Eq. (3).

**10.** **Eq (6) is unclear and the explanation below is confusing.**

**Response:** Thanks for the reviewer's comments. I will re-explain **Eq (6)** here and hopefully answer your questions. At this stage, the indicators for studying provincial differences mainly include coefficient of variation, Mean Log Deviation Index (MLD), Tyrell's index and Gini Coefficient, etc. However, when analyzing the above methods, most of them cannot decompose the provincial differences or do not consider the distribution of sample subgroups when decomposing, so they all have certain defects in the application. To overcome the above difficulties, some researchers have used Dagum Gini coefficient to analyze provincial dynamic differences and evolutionary trends. In this paper, the Dagum Gini coefficient is utilized to measure the inter-provincial differences in the intensity of agricultural carbon emissions in China and decompose them to obtain the composition of the relative differences between inter-provincial regions.

$G=\sum_{j=1}^{k} \sum_{h=1}^{k} \sum_{i=1}^{C_{j}} \sum_{r=1}^{C_{h}} |y_{ji}-y_{hr}|/2C^{2}\bar{y}$（6）

In the equation, y_ji_ (y_hr_) denotes the agricultural carbon intensity of any provincial unit within the j(h) region, C is the number of sample provinces, is the national average of agricultural carbon intensity, k is the number of regional divisions, and C_j_(C_h_) is the number of provincial units within the j(h) region.

**11.** **Eqs (8-10) are also not clear, and many terms are not explained. The authors should define δ.**

**Response:** Thanks for the reviewer's comments. I'm going to re-explain **Eqs (8-10)** here and hopefully answer your questions:

To test hypothesis H3, a mediating effects model was used to test the mediating effects of technological progress, the government's ability to allocate financial resources, and the mediating effects of the combination of the two, respectively.

$RIF\left\{ M_{n};v^{DG}(F_{Y}) \right\}=\gamma_{0}+\gamma_{n}X+\lambda_{2}Z+\emptyset+\mu$ （8）

$RIF\left\{ {ln}_{y_{it}},v^{DG}(F_{Y}) \right\}=k_{0}+\sum_{n=1}^{2} \varphi_{n}M_{n}+k_{1}X_{it}+k_{2}Z_{it-1}+\emptyset+\delta$(9)

$\beta_{1}=k_{1}+\sum_{n=1}^{2} o_{n}\gamma_{n}$(10)

In the equation, μ and δ are random disturbance terms, $M_{n}$ the mediating variable includes technological progress and financial resource allocation capacity. $\gamma_{0}$ and $k_{0}$ are intercept terms. $\gamma_{n}$and $\lambda_{2}$ are regression coefficients, $k_{1}$ denotes the direct effect of the core explanatory variables, and $\beta_{1}$ is the total effect of the impact of digital financial inclusion on the intensity of carbon reduction in agriculture and the gap in the intensity of carbon reduction in agriculture. $\varphi_{n}$ and $\gamma_{n}$ refer to individual mediating effects, and $\beta_{1}- k_{1}$ signifies the joint mediating effect. If $\gamma_{n}$ in equation (8) and $\varphi_{n}$ in equation (9) are both significant, it can be shown that digital financial inclusion can reduce agricultural carbon emission intensity and Dagum Gini coefficient through the mediating variable $M_{it}$. Conversely, if they are not significant, the opposite is true.

**12.** **Section 3.2.4 is unclear, and the RIF-Blinder-Oaxaca decomposition method needs to be explained and how to decompose it.**

**Response:** Thanks for the reviewer's comments. The RIF-Blinder-Oaxaca decomposition methodology and how it is done has been re-explained in the text as follows:

The regional agricultural carbon emission intensity gap decomposition method combines the implications of the regression of the regrouping influence function (RIF) method, which decomposes the total interprovincial agricultural carbon emission intensity differences into structural and characterization effects by constructing a counterfactual distribution function, so as to obtain the specific contribution of each explanatory variable to the total differences, and extends it to RIF regressions of the Gini coefficient and the Atkinson's index. Among them, the structural effect is determined by the difference in total agricultural carbon emissions between different inter-province caused by the different shares of agricultural GDP; the characterization effect is caused by the differences in the individual characteristics of these two groups, such as the basis of socio-economic development and differences in human capital.

**13.** **It is unclear whether Table 3 was produced from RIF models and which equations were used.**

**Response:** Thanks for the reviewer's comments. The results in Table 3 are from Equation $RIF\left\{ {lny}_{it},v^{D-G}(F_{Y}) \right\}=\beta_{0}+\beta_{1}X_{it-1}+\beta_{2}Z_{it-1}+\emptyset+\eta+\varepsilon_{it}$ (1) and are the results of the baseline regression using the regression of the reunification impact function (RIF) method.

**13.** **Table 5 is derived from quantile regression, but the explanation in the methodology section does not make sense. All the coefficients are constant and do not vary according to quantile level.**

**Response:** Thanks for the reviewer's comments. The results in Table 3 are from Equation $RIF\left( lny_{it} | \emptyset;\gamma;q_{r} \right)=B\tilde{X}\tilde{Z|}\emptyset+\varepsilon_{it}$ (3). And this paper examines the effect of digital financial inclusion on agricultural carbon emission intensity at different quantile points through unconditional quantile regression, and the results are shown in Table 5. The absolute value of the regression coefficient of the digital financial inclusion index gradually decreases as the quantile points rise, and the regression coefficient is no longer significant when it is at the 90th quantile point. This means digital financial inclusion strongly impacts high agricultural carbon intensity provinces. The effects of the three dimensions and the total index converge, with the breadth of coverage having a more significant negative effect. Thus, hypothesis H2a is verified. Figure 4 shows the quantile effect of digital financial inclusion on the D-Gini coefficient. The white color indicates that the significance level is not reached, and the black color indicates that the significance test is passed. That is, digital financial inclusion can significantly reduce the regional agricultural carbon intensity gap.

**Reviewer #4:**

**1. The title solely addresses the carbon emission gap, without acknowledging carbon emission intensity. Nevertheless, it is crucial to note that the text extensively covers the aspect of carbon emission intensity as well. Hence, I suggest authors focus on the carbon emission intensity may be more suitable. You can refer to the following papers related to carbon emission intensity: Does digital inclusive finance affect urban carbon emission intensity: Evidence from 285 cities in China. Cities**

**Response:** Thanks for the reviewer's comments. Your feedback was constructive, as it specifically highlighted issues with the title of our paper. The text does make extensive reference to the carbon intensity aspect, and we have made minor adjustments to the title to address this and your comments: Can digital financial inclusion converge the regional agricultural carbon emissions intensity gap? Then, we read the reference provided by you and found it to be instrumental in enhancing our paper. Therefore, we have cited it in the manuscript. For details, please refer to the revised manuscript.

**2. The revision of provincial differences into provincial differences is recommended.**

**Response:** Thanks for the reviewer's comments. Your comment is very useful for improving our articles' quality, and we have already changed "regional differences" to "provincial differences".

**3. The conflicting arguments regarding carbon intensity and its regional disparities may potentially perplex the readers.**

**Response:** Thanks for the reviewer's comments. The carbon intensity of a region's agriculture refers to the total agricultural carbon emissions ratio to the region's agricultural gross domestic product (agricultural GDP). The structure of agricultural cultivation, the scale of agricultural production, the level of mechanization, and the level of commercialization of agricultural products in each region will all affect the total agricultural GDP of a region. The different amounts of fertilizers, pesticides, agricultural films, etc., used in agriculture will lead to different total agricultural carbon emissions in each region. Therefore, the two's ratio is subject to large regional differences.

**4. The methodology section suggests emphasizing the merits of the RIF method in coping with carbon intensity gap measurements.**

**Response:** Thanks for the reviewer's comments. We have already emphasized the merits of the RIF approach in responding to carbon intensity gap measurements in the methodology section, as described below:

The general measurement method cannot be easily extended to non-central locations, and only observes the average treatment effect of digital inclusion, ignoring the impact of digital inclusion on regional heterogeneity of interprovincial agricultural carbon emission intensity, such as the impact of digital inclusion on high agricultural carbon emitting provinces (the upper tail) versus low agricultural carbon emitting provinces (the lower tail), which are often the two groups that have the most direct role in the effect of the carbon agricultural emission reduction effect of digital inclusion Firpo et al. (2018) further expand on the technique of conditional quantile regression with the proposed regression of the regrouping influence function (RIF), which assesses the effect of changes in the distribution of the explanatory variables on the quantiles of the unconditional (marginal) distribution of the outcome variable, and which achieves an essential breakthrough from focusing on the decomposition of differences in the means to the decomposition of differences in the entire distribution, and is capable of give the degree of influence of each independent variable, better avoiding the endogeneity problem arising from omitted variables. Based on this, to test hypotheses H1 and H2, we used the regression of the reaggregated impact function (RIF) method to examine the impact of digital financial inclusion on inter-provincial changes in agricultural carbon intensity and variation in China.

**5. The presence of numerous errors is evident. Such as "Error! Reference source not found."**

**Response:** Thanks for the reviewer's comments. We've re-colored the language in the article to avoid a recurrence of the problem you described.

**6. The excessive inclusion of Chinese references in the paper does not align with the criteria set by English journals. I suggest authors cite more papers from international journals. Some papers maybe useful for your revision: Seeing green: how does digital infrastructure affect carbon emission intensity? Energy Economics. Reducing carbon emissions: Can high-speed railway contribute? Journal of Cleaner Production.**

**Response:** Thanks for the reviewer's comments. The problem you describe is in the article, and the references have been updated. Also, the article you recommended was beneficial, and we have cited it.

**7. The language expression and textual logic still have ample room for improvement. Take the first paragraph of the introduction for example, what does "The former establishment" refer to? What does "the 20-year period" mean? What does "Agriculture and land-use change account for a quarter of total global emissions of greenhouse gases" mean? What does "LCA" refer to?**

**Response:** Thanks for the reviewer's comments. We have polished the language of the article and hope that the issues you raised will be improved. We have redacted statements with ambiguity. For details, please refer to the revised manuscript.
