# Supplementary material for: Can digital financial inclusion converge the regional agricultural carbon emissions intensity gap?
Source: PLoS One. 2024 Jul 16;19(7):e0307328. doi: 10.1371/journal.pone.0307328 (PMC11251634; doi:10.1371/journal.pone.0307328)
Supplement: Attachment — Submitted filename: Response to Reviewers.docx [file pone.0307328.s003.docx]

Dear Editors and Reviewers:

Thank you for your letter and the reviewers' comments concerning our manuscript entitled "Can digital financial inclusion converge the regional agricultural carbon emissions intensity gap?" Those comments are all valuable for revising and improving our paper and help guide our research.

We have accepted the reviewers' important recommendations and strive to make the best effort to improve the manuscript. Specifically, we have made some revisions to the manuscript, but these changes will not affect this paper's leading content and framework. Revised portions are marked with red fonts in the paper. The main corrections in the paper and the responds to the reviewers' comments are as flowing.

We appreciate for Editors/Reviewers' warm work earnestly, and hope that the correction will meet with approval.

Once again, thank you very much for your comments and suggestions.

Sincerely yours,

Lingzhi Tan

**Responds to the reviewer's comments:**

**Reviewers' comments:**

**Reviewer #3:**

**1.Some citations remain unedited, such as in Section 2.3 (e.g., Suhrab M et al. (2024), Chen YB et al. (2022) [43]).**

**Response:** Thanks for the reviewer's comments. The problem you are referring to is the typography that is needed according to the article. However, we have partially taken your comments and corrected them.

**2. The discussion on the contribution and research gap in this study. It should be highlighted in the last paragraph to underscore the paper's novelty. Additionally, Section 6.3 only outlines the purpose and advantages of the study; there is no clear identification of the research gap or the unique value this paper adds to the existing literature.**

**Response:** Thanks for the reviewer's comments. Your comments were very helpful, so we have made the following changes:

Research on the impact of finance on regional differences in carbon emissions has a long history. Whether the digital transformation of agriculture promoted by digital financial inclusion can effectively achieve the dual-carbon goal of agriculture in the region, and whether it can reduce the intensity of agricultural carbon emissions in neighboring regions by means of the convenience, inter-temporality and low-cost characteristics of digital financial inclusion has not yet been validated, and even less has been demonstrated to show how digital financial inclusion can achieve convergence of the inter-regional disparity in agricultural carbon emissions. In view of this, this paper utilizes the panel data of 30 provinces in China (excluding Tibet, Hong Kong, Macao and Taiwan) from 2010 to 2020, and combines with Peking University's digital financial inclusion database, etc., in order to verify the impact of digital financial inclusion on the gap of inter-regional agricultural carbon emission intensity and the mechanism of its effect. The innovations in this study are as follows.

1. It examines the impact of digital financial inclusion on the intensity of agricultural carbon emission reduction. It investigates whether it can facilitate the reduction of agricultural carbon emissions and the convergence of the regional gap in agricultural carbon emission intensity.

2. It elucidates the impact mechanism of digital financial inclusion from financial resource allocation efficiency and technological innovation, offering a novel perspective for analyzing the path of convergence of the regional gap in carbon emission intensity through digital financial inclusion.

3. It incorporates the urban-rural income disparity. It comprehensively analyzes how digital financial inclusion influences the gap in agricultural carbon emission reduction intensity from the perspective of changes in the urban-rural income disparity. However, we also acknowledge some limitations.

However, we also admit limitation: Due to the data availability, this paper only analyzes the values of agricultural and regional carbon emission intensity Dagum Gini coefficient from 2010 to 2020, the effects of digital financial inclusion on them, and the underlying mechanisms. Therefore, the timeliness of the data may be inadequate, and we hope that future research will be carried out with more updated data sources. So, the time series of data can be added in future research to enhance the effectiveness of the results.

**3.The methodology is unclear and contains unusual notations. For instance, ^− () represents the Dagum Gini coefficient whixh doest not make any sense here, and some notations lack italic formatting. Equations used in this study are not convincing, and there is a missing explanation of the unconditional quantile regression model.**

**Response:** Thanks for the reviewer's comments.

About the methodology. **Firstly,** this paper adopts the regression method of regrouping influence function (RIF) to elucidate the impact of digital financial inclusion on the intensity and variation of agricultural carbon emissions between provinces in China. The specific procedures are as follows: first, the conditional mean function, quantile points and Dagum Gini coefficient are used to conduct RIF regression analysis. Specifically, the conditional mean function is used for RIF regression analysis to test whether digital financial inclusion affects the change of interprovincial agricultural carbon emission intensity; the unconditional quantile regression model is constructed to consider the change of interprovincial agricultural carbon emission intensity at different quantile points; second, the mediation effect model is used to analyze the mechanism of the impact of digital financial inclusion on the regional disparity of agricultural carbon emission intensity; third, the RIF- Blinder-Oaxaca model to construct a counterfactual distribution function to decompose the influencing factors of the interprovincial agricultural carbon emission reduction intensity gap; and the moderating effect model is used to verify the influencing effects of the moderating variables. **Second,** the mediating effects model is used to test the mediating effects of technological progress and the government's ability to allocate financial resources, as well as the mediating effects of the combination of the two, respectively. **Lastly,** the moderating effect of the urban-rural income gap on the convergence of regional agricultural carbon emission reduction intensities was examined, while the RIF-Blinder-Oaxaca decomposition method was used to seek the main factors affecting the regional agricultural carbon emission reduction intensity gap.

Regarding the uncommon symbols and italics in the text, we have changed them in the text, thank you for your comments.

The unconditional quantile regression model is interpreted as follows:
The general measurement method cannot be easily extended to non-central locations, and only observes the average treatment effect of digital financial inclusion, ignoring the impact of digital financial inclusion on regional heterogeneity of inter-provincial agricultural carbon emission intensity, such as the impact of digital financial inclusion on high-agricultural carbon-emitting provinces (upper tail) versus low-agricultural carbon-emitting provinces (low-tail), which tend to be the two groups that are the most direct role of digital financial inclusion in carbon agricultural emission reduction effects groups.Firpo et al. (2018) further add an extension to the technique of conditional quantile regression with the proposed regression of the regrouping influence function (RIF), which assesses the effect of changes in the distribution of the explanatory variables on the quantiles of the unconditional (marginal) distributions of the outcome variables, and is able to achieve the important breakthrough of overstepping from focusing on decompositions of differences in means to decompositions of differences in the entire distribution, as well as being able to give the degree of influence of each independent variable, better avoiding the endogeneity problem arising from omitted variables. Based on this, the regression method of regrouping influence function (RIF) is used to elucidate the impact of digital inclusive finance on the changes of agricultural carbon emission intensity and differences between provinces in China.Firpo et al (2018) argued that the regression method of regrouping influence function can better avoid the problem of endogeneity generated by the omitted variables, and is currently mostly used in the income disparity, rich-poor disparity measurement and so on. The specific procedures are as follows: First, the conditional mean function, quantile points and Dagum Gini coefficient are used for RIF regression analysis. Specifically, the conditional mean function is used for RIF regression analysis to test whether digital financial inclusion affects the change of interprovincial agricultural carbon emission intensity; the unconditional quantile regression model is constructed to consider the change of interprovincial agricultural carbon emission intensity at different quantile points; second, the mediation effect model is used to analyze the mechanism of the impact of digital financial inclusion on the regional disparity of the agricultural carbon emission intensity; and third, the RIF- Blinder-Oaxaca model to construct a counterfactual distribution function to decompose the influencing factors of the interprovincial agricultural carbon emission reduction intensity gap; and the moderating effect model is used to verify the influencing effects of the moderating variables.

**4.In Table 4, the differences between columns 1-2, 4-5, and 7-8 are unclear. How are they different.**

**Response:** Thanks for the reviewer's comments. As explained earlier, To avoid your question, the explanation has now been reintroduced in the text as follows: The difference between them is that columns (1) (4) (7) are the conditional mean model without control variables, while columns (2) (5) and (8) are the regression results after adding the control variables.
